# Human transforming growth factor β type I receptor in complex with kinase inhibitor SB505124

**DOI:** 10.1107/S2053230X24010094

**Published:** 2024-10-23

**Authors:** Jhon A. Rodriguez Buitrago, Maréne Landström, Magnus Wolf-Watz

**Affiliations:** a Department of Chemistry Umeå University Linnaeus vag 10 901 87Umeå Sweden; b Department of Medical Biosciences Umeå University 901 85Umeå Sweden; Bristol-Myers Squibb, USA

**Keywords:** TGF-β type I receptor, inhibition, intracellular domain, prostate cancer, osteoblast differentiation

## Abstract

The crystal structure of the intracellular domain of transforming growth factor β type I receptor (TβR1) in complex with the competitive inhibitor SB505124 is presented. The study provides insights into the structure and function of TβR1 in complex with SB505124, and as such offers molecular-level understanding of the inhibition of this critical signalling pathway. The potential of SB505124 as an avenue for therapy in cancer treatment is discussed on basis of the results.

## Abstract 2

This study presents the crystal structure of transforming growth factor β type I receptor (TβR1) in complex with SB505124, highlighting the specific contacts that SB505124 makes with TβR1 and comparing these interactions with those of SB431542.

## 1. Introduction

The transforming growth factor β (TGF-β) superfamily encompasses a broad spectrum of members, including TGF-βs and bone morphogenetic proteins (BMPs), that regulate diverse cellular processes such as growth, differentiation and apoptosis (Heldin *et al.*, 2009 ▸; Derynck & Miyazono, 2007 ▸). Activation of TGF-β signalling involves specific type I and type II serine/threonine kinase receptors, ultimately leading to the phosphorylation of Smad proteins, notably Smad2 and Smad3, to orchestrate gene expression (Morikawa *et al.*, 2016 ▸). TGF-β is a key regulator in various types of cancer, exerting its effects through the intracellular domain (ICD) of TGF-β receptor I (TβRI; Boguslawska *et al.*, 2019 ▸). *In vitro* studies employing small-molecule inhibitors targeting intracellular signalling pathways have proven to be invaluable in dissecting the molecular pathways enabling cancer progression. These inhibitors offer a promising avenue for therapeutic intervention, presenting a chemical biology approach to modulating the aberrant signalling cascades implicated in cancer pathogenesis (Jones *et al.*, 2009 ▸). Specifically, inhibitors targeting the TGF-β signalling pathway hold potential not only in unravelling its intricate interactions with other signalling pathways, but also in the development of novel therapeutic strategies for a wide array of cancer types (Morikawa *et al.*, 2016 ▸). In this manuscript, we elucidate the crystal structure of the intra­cellular domain of TβR1 (ICD) in complex with the inhibitor SB505124 [2-(4-(benzo)[d][1,3]dioxol-5yl)-2-*tert*-butyl-1H-imidazol-5-yl)-6-methylpyridine], shedding light on the regulatory mechanisms of TGF-β signalling and its implications in cancer biology. The molecule SB431542 {4-[5-(1,3-benzodioxol-5-yl)-4(pyridin-2-yl)-1H-imidazol-2-yl]benzamide}, a TGF-β type I receptor inhibitor, has been crystallized in complex with ICD (PDB entry 3tzm; Ogunjimi *et al.*, 2012 ▸) and has been reported to be a potent suppressor of osteoblastic differentiation in human bone marrow mesenchymal stem cells (hBMSCs). Previous studies (Almuraikhi, 2023 ▸) suggested that SB505124 inhibits osteoblast differentiation of hBMSCs, as shown by reduced alkaline phosphatase activity, *in vitro* mineralization and downregulation of osteoblast-associated gene expression, along with other genes linked to osteoblast-related signalling pathways (Almuraikhi, 2023 ▸). This suggests the potential use of SB505124 in bone diseases characterized by increased bone formation and mineralization, also including some human cancers, such as prostate cancer, where high expression of TβRI ligands is known to play a key role in tumour progression and indicates a poor prognosis for the patient (Mu *et al.*, 2011 ▸). It holds promise as an innovative therapeutic approach for bone disorders and may also have applications in the treatment of cancer and fibrosis (Almuraikhi, 2023 ▸). Here, we present the structure of the TβR1–SB505124 complex, together with a detailed discussion of the function of the macromolecular complex, along with comparisons with the related structure obtained using the related compound SB431542 (Ogunjimi *et al.*, 2012 ▸). Collectively, this new structure and the previous characterization of SB505124 (Morikawa *et al.*, 2016 ▸) provide comprehensive insights into the action of this TβR1 inhibitor.

## 2. Materials and methods

### 2.1. Macromolecule production

Protein expression was performed at Lund Protein Production Platform (LP3). The coding sequence of the human TβR1 intracellular domain (ICD; residues 200–503) was synthesized and codon-optimized for expression in insect cells and cloned using BamHI/NotI into pFastBac1 by GenScript. The donor plasmid His-TEV-TβR1_pFastBac1 (pLP 743) was used to make a recombinant bacmid according to the instructions for the Bac-to-Bac system provided by the manufacturer (Invitrogen) using DH10MEmBacY cells (Geneva Biotech) (Table 1 ▸). Baculovirus generation was performed using Sf9 insect cells (Invitrogen), SFX medium (Cytiva) and baculoFECTIN II (Oxford Expression Tech­nologies). The resulting virus was then used to generate baculovirus-infected Sf9 cells (BIIC; Wasilko *et al.*, 2009 ▸). The harvested BIIC were stored in aliquots at −80°C until use. The previously prepared BIIC stock was then used to infect large-scale cultures for expression using Sf9 cell lines and a multiplicity of infection (eMOI) of 0.5. After analysis of the first expression using immunoblotting, it was determined that the optimal expression on a larger scale needed to be adjusted by allowing an extra day prior to harvesting (four days post-infection).

#### 2.1.1. Infection

Sf9 insect cells were diluted to 1 × 10^6^ cells ml^−1^ for each expression flask. Expression was performed in 1 l cultures in SFX medium. The cells were infected with the BIIC stock virus with an eMOI of 0.5. The cultures were incubated (100 rev min^−1^, 27°C) until harvesting (three or four days). 1 ml samples were taken after infection and the pellets were stored at −20°C until analysis by Western blotting. Additionally, a small sample was also used to monitor fluorescence, cell count and viability before harvesting. A cellular extract (lysate) was prepared from the cell pellets by sonication in 0.25 ml 50 m*M* sodium phosphate buffer pH 8, 300 m*M* NaCl. The lysate was then centrifuged at 20 000*g* and 4°C for 45 min to clarify the soluble fraction (the supernatant). The samples were then run on an AnykD Criterion TGX precast gel with 18 wells (Bio-Rad) at 100 V for 10 min followed by 200 V for 35 min. A Western blot was performed directly after the run using RTA transfer packs with PVDF membrane (Bio-Rad), and with mouse anti-6×His (BD) as the primary antibody (1:20 000) and goat anti-mouse IgG–peroxidase (Bio-Rad) as the secondary antibody (1:300 000). Visualization of the signal was performed using ECL reagent (Bio-Rad) and a ChemiDoc MP System (Bio-Rad). The cell pellet was resuspended in 50 ml lysis buffer (20 m*M* Tris pH 8.0, 300 m*M* NaCl) in the presence of protease inhibitors (one tablet of cOmplete EDTA-free protease-inhibitor cocktail per 50 ml; Sigma–Aldrich). After sonication (nine cycles of 20 s at an amplitude of 60% followed by a 10 s pause on ice using a Vibra-Cell VCX130, Sonics & Materials, USA) for cell disruption and subsequent centrifugation (15 min, 10 000*g*, 4°C) to remove cell debris, ICD was found in the soluble fraction. The His_6_-tag-containing ICD was purified by affinity chromatography on an ÄKTAprime FPLC system (GE Healthcare, Freiburg, Germany) using a 5 ml HisTrap HP column (GE Healthcare). The bound protein was eluted using a linear imidazole gradient (0–0.5 *M*) in five column volumes (CV). Selected ICD-containing fractions were combined for incubation with TEV protease at a ratio of 1:30 and were then dialyzed against 20 m*M* Tris pH 8.0, 40 m*M* NaCl at 4°C for 16 h. Dialyzed samples were again loaded onto a HisTrap HP column (GE Healthcare) to remove the cleaved His-tag. The ICD-containing flowthrough was loaded onto a 5 ml HiTrap Q HP column (GE Healthcare) and elution was performed using a linear NaCl gradient (0.04–1 *M*) in 5 CV. Selected ICD-containing fractions were concentrated by ultrafiltration using a 10 kDa cutoff membrane (Amicon Ultra-15, Merck) and were further purified by gel filtration on a Superdex 75 26/60 column (GE Healthcare) using 20 m*M* Tris pH 8.0, 300 m*M* NaCl, 1 m*M* dithiothreitol. Purified ICD was obtained with a yield of 5 mg per litre of culture. A high degree of purity was confirmed for ICD by the observation of a single band on a 12% SDS–PAGE gel.

### 2.2. Crystallization

Initial crystallization trials were carried out at room temperature with Crystal Screen and Crystal Screen 2 from Hampton Research using the sitting-drop vapour-diffusion method. Crystallization droplets were formed using a nanodrop crystallization robot (Mosquito, SPT Labtech), with crystals of ICD being obtained after five days (Table 2 ▸).

The crystals were needle-shaped. Trials to optimize the crystal quality by varying the precipitant concentration or the pH did not result in improved diffraction.

### 2.3. Data collection and processing

ICD–SB505124 crystals were harvested using a nylon loop (Hampton Research) and were soaked in reservoir solution containing 23%(*w*/*v*) PEG 3350 and 3%(*v*/*v*) glycerol at pH 5.6 prior to flash-cooling in liquid nitrogen. A total of 900 images were collected using the oscillation method. Data-collection and processing statistics are summarized in Table 3 ▸. Reflection-image processing was performed using *autoPROC* (Vonrhein *et al.*, 2011 ▸), which uses *XDS* (Kabsch, 2010 ▸) for integration, *POINTLESS* (Evans, 2006 ▸) from the *CCP*4 suite (Agirre *et al.*, 2023 ▸) for space-group determination, *AIMLESS* (Evans & Murshudov, 2013 ▸; Agirre *et al.*, 2023 ▸) for scaling and merging and *STARANISO* (Tickle *et al.*, 2018 ▸) for anisotropic scaling and correction of diffraction data; the data results were delivered by *CCP*4 Cloud (Krissinel *et al.*, 2022 ▸).

### 2.4. Structure solution and refinement

The initial phases were obtained by molecular replacement using *MrBUMP* (Keegan & Winn, 2008 ▸), executing *Phaser* (McCoy *et al.*, 2007 ▸) and using the atomic coordinates of the transforming growth factor β type I receptor activin-like kinase 5 (ALK5; PDB entry 2wot; Goldberg *et al.*, 2009 ▸) as a search model. Refinement was performed by alternating rounds of *REFMAC*5 (Murshudov *et al.*, 2011 ▸) and manual adjustments in *Coot* (Emsley *et al.*, 2010 ▸). Refinement statistics are given in Table 4 ▸. The diffraction data and coordinates have been deposited in the Protein Data Bank (PDB; Berman *et al.*, 2002 ▸) as entry 9f6x. Representations of the structures were generated with *USCF Chimera* (Pettersen *et al.*, 2021 ▸).

## 3. Results and discussion

### 3.1. Structure of the ICD–SB505124 complex

**Figure 1:**
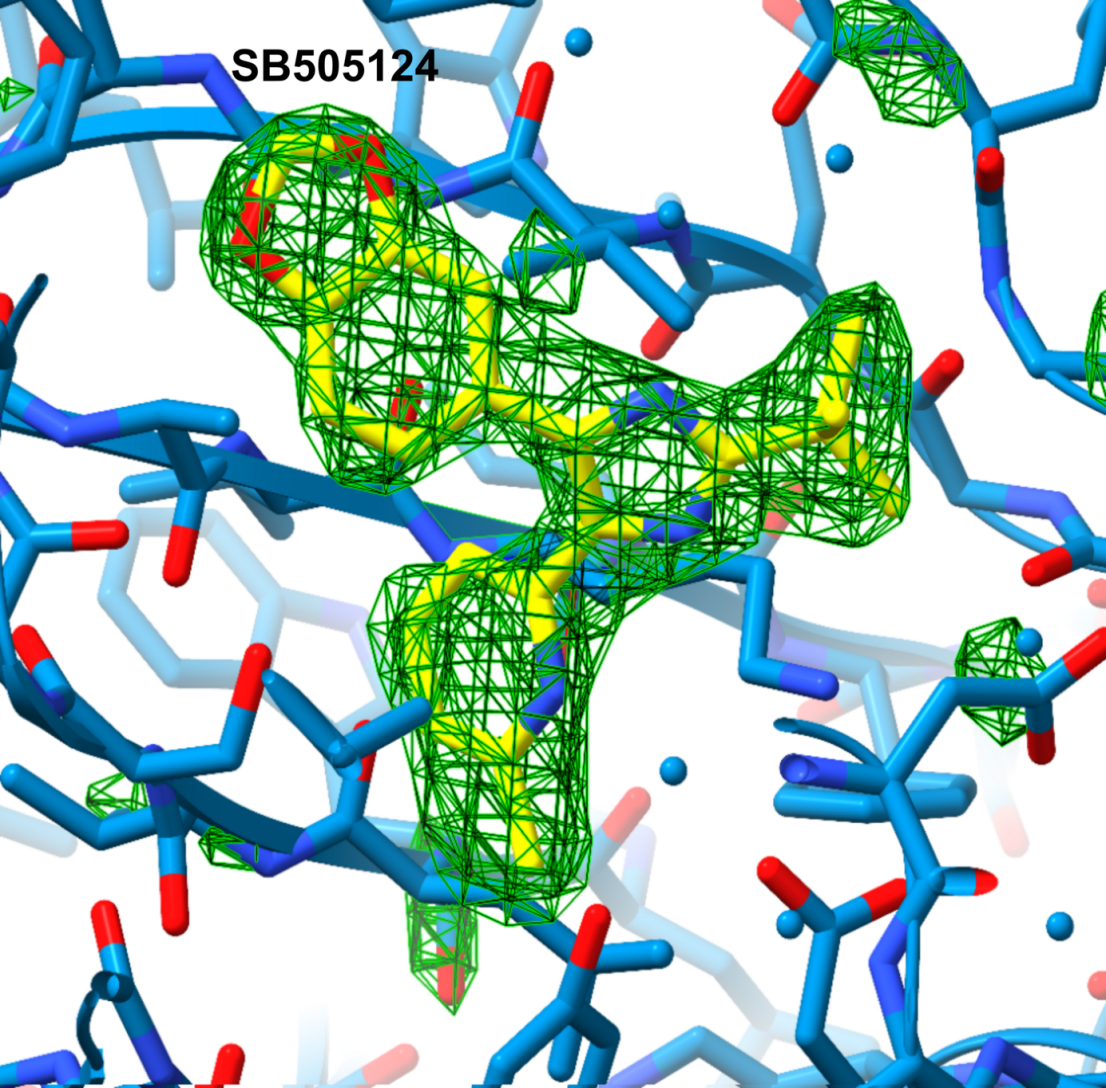
The ICD–SB505124 complex. *F*_o_ − *F*_c_ electron density (green) contoured at 3 r.m.s.d showing evidence for the bound ligand. Omit maps were generated by removing the ligand from the structure and running three cycles of gradient energy minimization and *B*-factor optimization in *Phenix* 1.21.1-5286 (Liebschner *et al.*, 2019 ▸) to minimize model bias.

**Figure 2:**
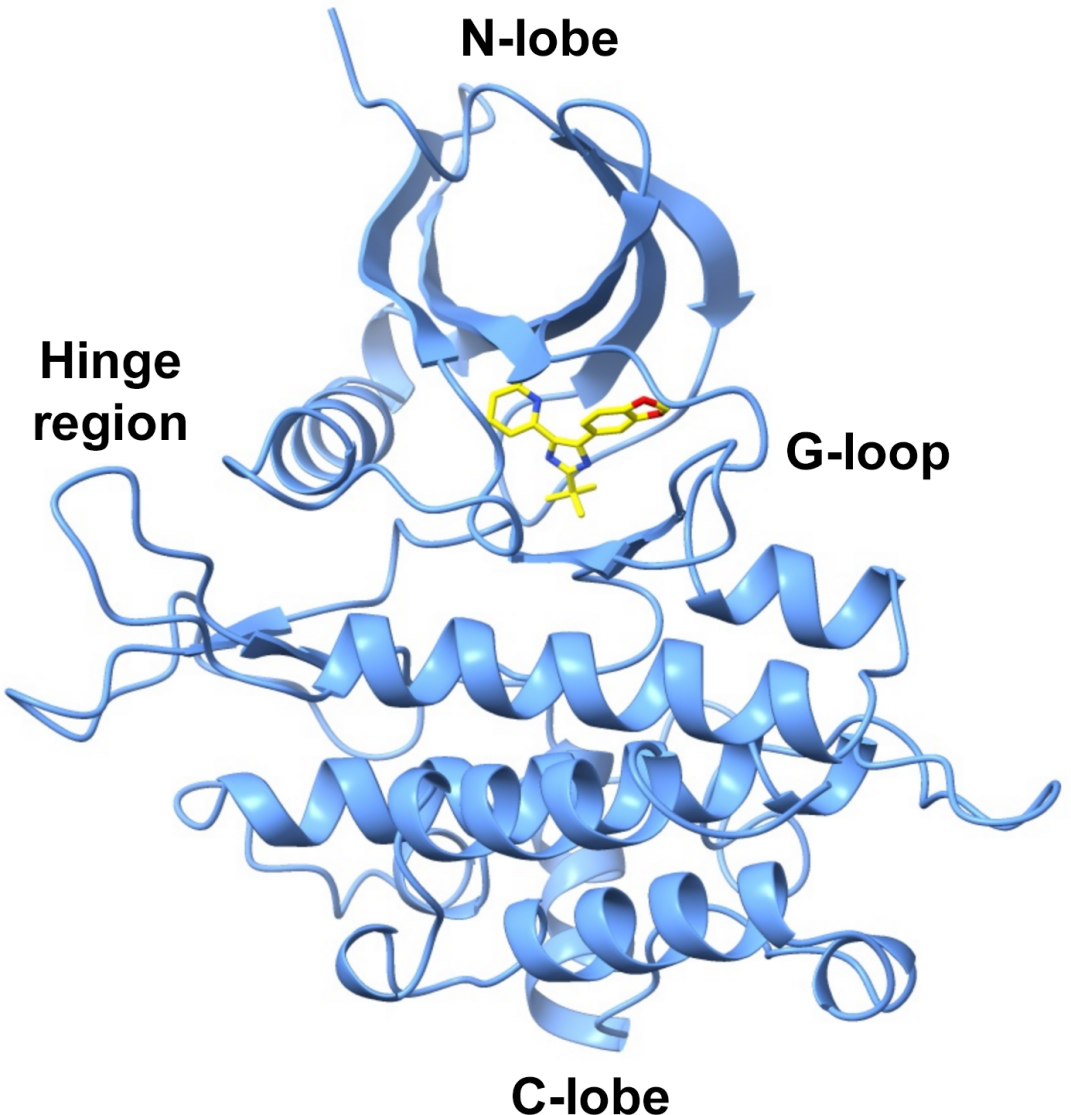
Crystal structure of the ICD–SB505124 complex. The structure of the ICD kinase domain is shown in blue and SB505124 is shown as yellow sticks occupying the ATP-binding cleft between the N- and C-lobes of the kinase.

**Figure 3:**
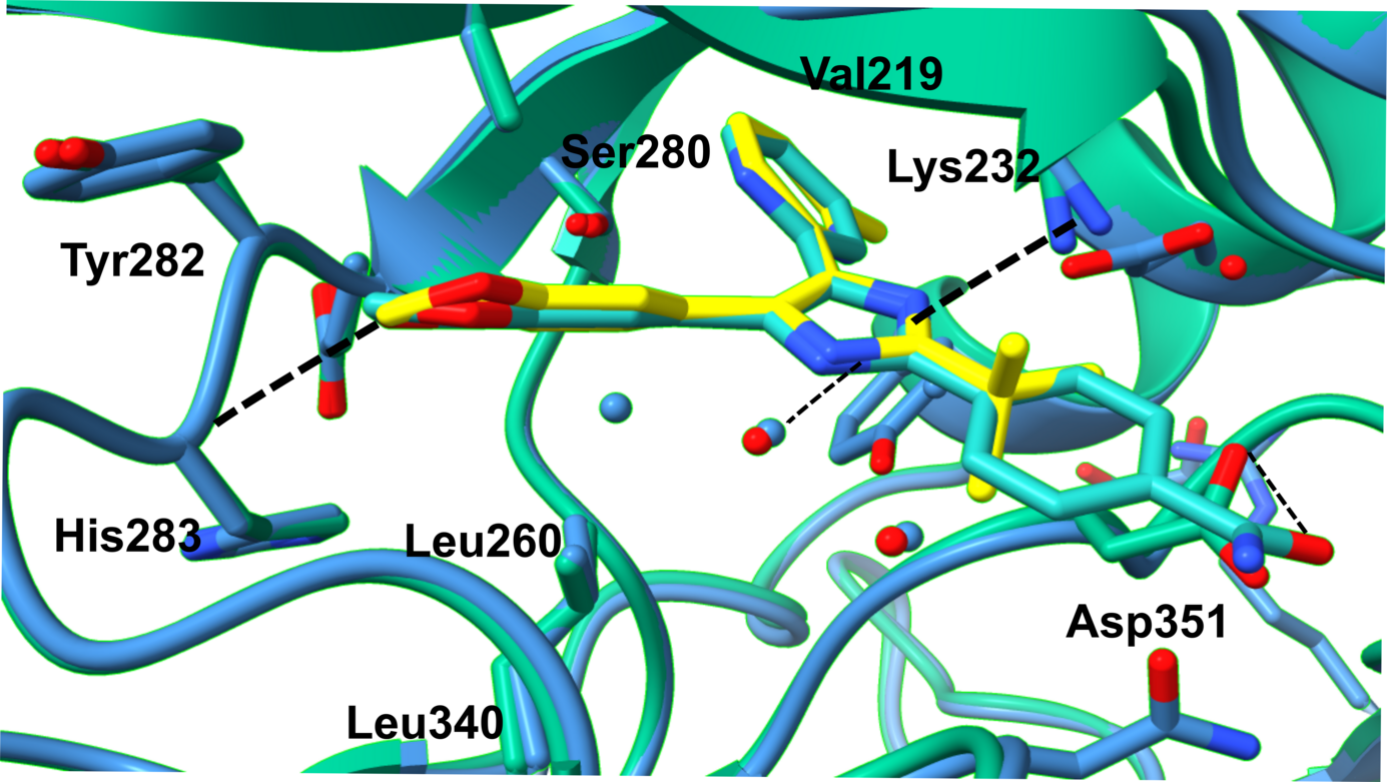
Close-up view of the overlaid structures of the ICD–SB505124 and ICD–SB431542 complexes, showing the compound interactions in the ATP-binding cleft and the residues contacting either SB505124 or SB431542. Colour code: ICD–SB505124 (PDB entry 9f6x), blue and yellow; ICD–SB431542 (PDB entry 3mtz), emerald and jade. Hydrogen bonds are shown as black dashed lines.

**Figure 4:**
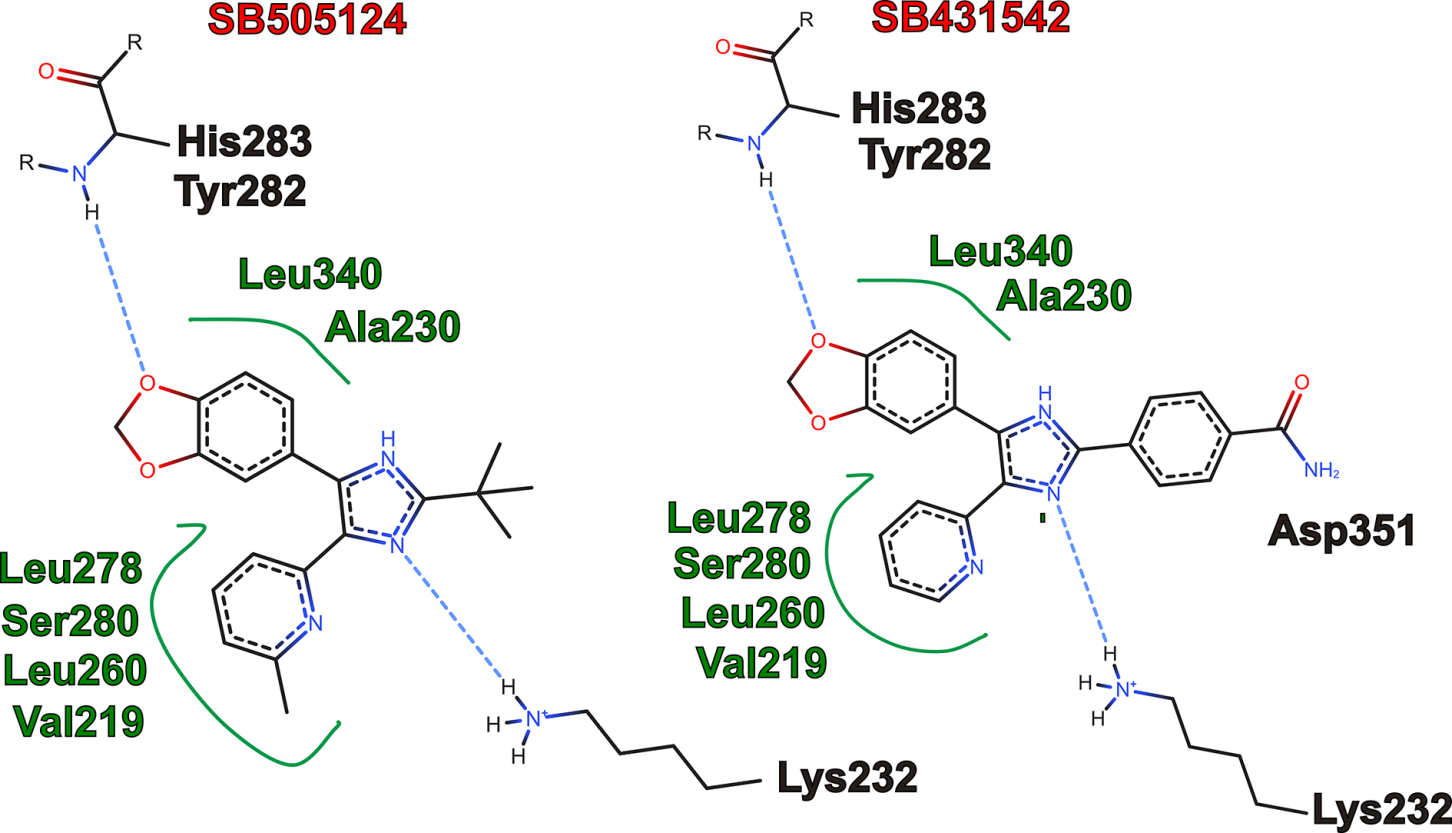
Interaction networks in 2D views of the ICD–SB505124 (left) and ICD–SB43154 (right) complexes.

We co-crystallized SB505124 bound to the transforming growth factor-β type I receptor (TβRI) intracellular kinase domain (ICD; residues 200–503; Tables 1 ▸ and 2 ▸). ICD crystallized with symmetry consistent with space group *P*2_1_2_1_2_1_, with one monomer in the asymmetric unit (Table 3 ▸). Initial phases were obtained by molecular replacement using the structure of the TβRI ALK5 (PDB entry 2wot; 100% amino-acid sequence identity) as a search model. The resulting electron-density map allowed identification of the SB505124 molecule bound to ICD (Fig. 1 ▸). The final ICD model, refined at 2.68 Å resolution with an *R*_cryst_ of 21% and an *R*_free_ of 25%, displayed good geometry (Table 4 ▸). ICD exhibits the classic kinase domain organization with a small N-terminal lobe and a larger C-terminal lobe (Fig. 2 ▸). The ICD activation loop adopts the extended conformation characteristic of active protein kinases (G-loop; Fig. 2 ▸). The TβRI–SB505124 complex closely resembles the TβRI kinase conformation observed in the TβRI–SB431542 complex (Ogunjimi *et al.*, 2012 ▸). SB505124 and SB431542 are both TGF-β signalling inhibitors but differ in their structural properties and selectivity. SB505124 (log*P* = 4.3), with its pyridine-benzamide framework, is specifically designed to inhibit ALK5 and is more hydrophobic than SB431542 (log*P* = 2.7), which may affect its solubility and interaction with receptors (Inman *et al.*, 2002 ▸). Log*P* values were obtained from PubChem (Kim *et al.*, 2016 ▸). This increased hydrophobicity impacts its distribution and bioavailability in biological membranes (Laping *et al.*, 2002 ▸). SB505124 has been demonstrated to be more potent than the ALK5 inhibitor SB431542. Comparative studies using a luciferase assay with the CAGA12–luciferase reporter construct have shown that SB505124 exhibits greater potency, consistent with reported IC_50_ values of 47 ± 5 n*M* for SB505124 and 94 n*M* for SB431542 in *in vitro* phosphorylation assays of immobilized Smad3 (Callahan *et al.*, 2002 ▸). Furthermore, while SB431542 exhibits cellular toxicity at concentrations of 100 µ*Min vivo*, SB505124 does not show toxicity at concentrations of up to 100 µ*M*, indicating better tolerability and the potential for effective inhibition of ALK4, ALK5 and ALK7 in *in vivo* models (B. A. Olson, M. Spengler & K. Salyers, unpublished work). From a structural perspective, the increased selectivity of SB505124 can be attributed to geometric differences between the compounds. On the other hand, the SB431542 structure contains imidazole, pyridine and benzamide rings, making it a broader-spectrum inhibitor that targets ALK4, ALK5 and ALK7. Our hypothesis is that SB431542 has a relatively flat structure with an unmodified dihydropyrimidinone ring, allowing it to bind more promiscuously across multiple ALK receptors. In contrast, the bulkier substituents in SB505124 are likely to restrict its accessibility to other ALK receptors, enhancing its selectivity. Molecular-docking studies and comparative modelling have also shown that SB505124 aligns more favourably within the ALK5 binding site compared with other ALK receptors, forming more stable interactions and supporting its structural selectivity (Sapitro *et al.*, 2010 ▸). Clear electron density was observed for SB505124 (Fig. 1 ▸), with a heterocyclic ring structure (pyridine, pyrimidine and benzene rings) similar to SB431542 (pyridine and benzodioxole rings); both of them act as a type I kinase inhibitor by occupying the ATP-binding region and adjacent hydrophobic sites near the hinge region, which is the linker between the N- and C-terminal kinase lobes (Fig. 2 ▸). ICD has a serine residue at the gatekeeper position (Ser280), allowing the hydrophobic pyridinyl ring of SB505124 and SB431542 to be accommodated within this pocket (Fig. 3 ▸). The position of the pyridinyl ring adjacent to the gatekeeper residue is further stabilized by hydrophobic interactions with Leu260 and Leu278. Leu260 and Ala230 contact both the pyridinyl and benzodioxole rings, which adopt a noncoplanar conformation (Fig. 3 ▸). The benzodioxole ring of SB505124 is sheltered by the hydrophobic residues Ala230, Leu260, Tyr282 (hinge region) and Leu340 (Fig. 4 ▸). Two hydrogen bonds link SB505124 with ICD: one is between the benzodioxole O atom of SB505124 and the amide N atom of His283 from the hinge region, a feature that is common to many ATP-competitive kinase inhibitors, while the second hydrogen bond is between Leu332 and the imidazole ring of SB505124, which is also stabilized by Val219 through hydrophobic interactions. Unlike SB431542, SB505124 does not contain a benzamide ring in its structure. The benzamide ring of SB431542, which is positioned between the glycine-rich sequence loop (residues 212–217; GKGRFG) and the activation segment, is coordinated partly through hydrogen bonding to Asp351 (Figs. 3 ▸ and 4 ▸). In conclusion, the amino acids involved in the stabilization of SB431542 and SB505124 are identical, with the difference lying in the benzamide ring region that is present in SB431542 but absent in SB505124. Notably, TβRI been found to undergo proteolytic cleavage in prostate cancer cells, and the ICD is translocated to the nucleus, where it promotes the invasion of cancer cells (Callahan *et al.*, 2002 ▸; Guo *et al.*, 2024 ▸). However, the clinical efficacy of SB505124 in the treatment of patients with prostate cancer requires further investigation.

## Supplementary Material

PDB reference: transforming growth factor β type I receptor, 9f6x

## Figures and Tables

**Table 1:** Macromolecule-production information

DNA source	Human
Forward primer	GGATCCGCCACC
Reverse primer	TAATAAGCGGCCGC
Cloning vector	pFastBac
Expression vector	Baculovirus expression vector system (BEVS)
Expression host	*Spodoptera frugiperda* (Sf9)
Protein sequence	MGSSHHHHHHSSGENLYFQGGTIARTIVLQESIGKGRFGEVWRGKWRGEEVAVKIFSSREERSWFREAEIYQTVMLRHENILGFIAADNKDNGTWTQLWLVSDYHEHGSLFDYLNRYTVTVEGMIKLALSTASGLAHLHMEIVGTQGKPAIAHRDLKSKNILVKKNGTCCIADLGLAVRHDSATDTIDIAPNHRVGTKRYMAPEVLDDSINMKHFESFKRADIYAMGLVFWEIARRCSIGGIHEDYQLPYYDLVPSDPSVEEMRKVVCEQKLRPNIPNRWQSCEALRVMAKIMRECWYANGAARLTALRIKKTLSQLSQQEGIKM

**Table 2:** Crystallization

Method	Vapour diffusion, sitting drop
Plate type	96-well Intelli-Plates
Temperature (K)	291
Protein concentration (mg ml^−1^)	10
Buffer composition of protein solution	20 m*M* Tris pH 8.0, 300 m*M* NaCl
Composition of reservoir solution	23%(*w*/*v*) PEG 3350, 3%(*v*/*v*) glycerol at pH 5.6
Volume and ratio of drop	200 nl, 1:1
Volume of reservoir (µl)	80

**Table 3:** Data collection and processing Values in parentheses are for the outer shell.

Diffraction source	ID30B, ESRF
Wavelength (Å)	0.87
Temperature (K)	100
Detector	EIGER2 9M
Crystal-to-detector distance (mm)	255.43
Rotation range per image (°)	0.2
Total rotation range (°)	180
Exposure time per image (s)	0.01
Space group	*P*2_1_2_1_2_1_
*a*, *b*, *c* (Å)	42.07, 79.85, 87.80
α, β, γ (°)	90, 90, 90
Mosaicity (°)	0.77–1.01
Anisotropic processing	
Resolution range (Å)	59.07–1.90
2σ operational diffraction limit (Å)	2.70
*STARANISO* difraction limits	1.79, 2.71, 2.46
Total No. of reflections	83424 (3703)
No. of unique reflections	12847 (642)
Completeness (overall/inner shell/outer shell) (%)	
Spherical	52.0/99.80/8.80
Ellipsoidal	92.40/99.80/7.50
Multiplicity	6.50 (5.8)
〈*I*/σ(*I*)〉	8.2 (1.9)
*R*_p.i.m._	0.06 (0.48)
*R*_meas_	0.15 (1.15)
CC_1/2_	0.99 (0.68)
Overall *B* factor from Wilson plot (Å^2^)	11
Isotropic processing	
Resolution range (Å)	59.07–2.02
2σ operational diffraction limit (Å)	2.83
Total No. of reflections	133166 (6333)
No. of unique reflections	19999 (970)
Completeness (%)	100 (100)
Multiplicity	6.7 (6.5)
CC_1/2_	0.997 (0.361)

**Table 4:** Structure solution and refinement Values in parentheses are for the outer shell.

Resolution range (Å)	10.87–2.68 (2.74–2.68)
Completeness (%)	96.20 (99.2)
σ Cutoff	2.02
No. of reflections, working set	8038 (510)
No. of reflections, test set	404 (22)
Final *R*_cryst_	0.19 (0.23)
Final *R*_free_	0.25 (0.39)
No. of non-H atoms	
Protein	2425
Ligand (SB505124)	25
Water	92
Glycerol	12
Total	2554
R.m.s. deviations	
Bond lengths (Å)	0.014
Angles (°)	2.7
Average *B* factor (Å^2^)	
Protein	20
Ligand (SB505124)	16
Water	28
Glycerol	24
Ramachandran plot†	
Most favoured (%)	94.30
Allowed (%)	5.70
PDB code	9f6x

† Model validation was performed with *MolProbity* (Chen *et al.*, 2010 ▸; Williams *et al.*, 2018 ▸).
